# Specific Intracellular Uptake of Herceptin-Conjugated CdSe/ZnS Quantum Dots into Breast Cancer Cells

**DOI:** 10.1155/2014/954307

**Published:** 2014-01-09

**Authors:** Seung-Jin Han, Pierson Rathinaraj, Soo-Young Park, Young Kyoo Kim, Joon Hyung Lee, Inn-Kyu Kang, Jong-Sik Moon, Jeffrey G. Winiarz

**Affiliations:** ^1^School of Applied Chemical Engineering, Kyungpook National University, Daegu 702-701, Republic of Korea; ^2^School of Advanced Materials Engineering, Kyungpook National University, Daegu 702-701, Republic of Korea; ^3^Department of Chemistry, Missouri University of Science and Technology, Missouri, MO 65409, USA

## Abstract

Herceptin, a typical monoclonal antibody, was immobilized on the surface of CdSe/ZnS core-shell quantum dots (QDs) to enhance their specific interactions with breast cancer cells (SK-BR3). The mean size of the core-shell quantum dots (28 nm), as determined by dynamic light scattering, increased to 86 nm after herceptin immobilization. The *in vitro* cell culture experiment showed that the keratin forming cancer cells (KB) proliferated well in the presence of herceptin-conjugated QDs (QD-Her, 5 nmol/mL), whereas most of the breast cancer cells (SK-BR3) had died. To clarify the mechanism of cell death, the interaction of SK-BR3 cells with QD-Her was examined by confocal laser scanning microscopy. As a result, the QD-Her bound specifically to the membrane of SK-BR3, which became almost saturated after 6 hours incubation. This suggests that the growth signal of breast cancer cells is inhibited completely by the specific binding of herceptin to the Her-2 receptor of SK-BR3 membrane, resulting in cell death.

## 1. Introduction

The development of noncytotoxic quantum dots (QDs) has attracted considerable interest as luminescence probes in biological and medical research because of their some unique optical and chemical properties [1], such as a tunable fluorescence wavelength according to size, a sharp and symmetrical fluorescence peak, strong and stable emission, high quantum yield, brightness, and photo stability [2–5]. QDs have several advantages over traditional dyes and fluorescent proteins used as imaging probes, such as tunable emission from visible to infrared wavelengths, broader excitation spectra, and high resistance to photo bleaching [6, 7]. On the other hand, the potential applications of QDs in biology and medicine are limited because of their toxic effects [8]. QDs contain toxic components, such as cadmium or lead. Cd^2+^ and Pb^2+^ can be released from QDs to kill the cells [9]. Recently, a number of techniques, such as a gold outer shell [10], targeted ligand-like peptide [11], proteins [12, 13], and polymer coating [14] have been developed to minimize the cytotoxicity of QDs. Consequently, many approaches for transforming QDs from hydrophobic to hydrophilic have been developed for a range of biomedical applications. Accordingly, researchers have also used noncytotoxic materials, such as polyethylene glycol (PEG) and polymaleic anhydride salt-1-tetradecene, to coat the surfaces of QDs [15].

Thus far, a range of surface coatings of QDs have been explored including the conjugation of mercaptoacetic acid [16], mercaptopropionic acid [17], mercaptobenzoic acid [18], and biocompatible and chemically functionalizable inorganic shells, such as silica or zinc sulfide [19]. These coatings can guarantee the water solubility of QDs but cannot enhance the biocompatibility significantly. Therefore, further coatings with suitable water-soluble organic ligand/biomolecules are necessary to enhance the biocompatibility of QDs. To that end, QDs have been linked covalently with biorecognition molecules, such as biotin [20], oleic acid [21], peptides [22], bovine serum albumin [23], transferrin [24], antibodies [25], and DNA [26].

Polymeric micelles have been studied extensively for the solubilization of hydrophobic drugs and bioactive agents because of their unique properties, including nanoscale size, high water solubility, high structural stability, high carrying capacity of hydrophobic agents, and easy introduction of functional moieties to the outer shell. The polymers generally leave the fluorescent properties of QDs unchanged but allow the introduction of other moieties to the QD surfaces [27]. CdSe/ZnS QD is a versatile core shell material with a wide band gap of 3.37 eV and a rather large exciton binding energy that makes the exciton state stable, even at room temperature. Zinc is a very important trace element in humans [28] and has been found to play an important part in many biological systems [29–32]. Therefore, CdSe/ZnS core-shell QDs are expected to be environmentally friendly and more suitable for bioimaging and cancer detection than CdSe QD.

In this study, breast cancer cells were targeted with herceptin-conjugated quantum dots for cancer therapy and diagnosis. Phospholipid-immobilized CdSe/ZnS core-shell quantum dots (QDs) were prepared by a coupling reaction of trioctylphosphine oxide-coated CdSe/ZnS core-shell quantum dots with carboxylic acid-terminated PEG and methoxy-terminated PEG. Herceptin was then introduced to the surface of the QDs (QD-Her) to enhance the antitumor effects of chemotherapeutic agents without increasing their toxicity [33–36]. The surface properties of the QDs and QD-Her were characterized by Fourier transform infrared (FT-IR) spectroscopy, electron spectroscopy for chemical analysis (ESCA), UV-Vis spectrometry, dynamic light scattering (DLS), and zeta potential measurements. To evaluate the cell compatibility and cytotoxicity of the QDs and QD-Her, human breast cancer cells (SK-BR3) were cultured in the presence of Q-dots. The intracellular uptake of QD-Her to the cells was also observed by confocal laser scanning microscopy (CLSM).

## 2. Materials and Methods

Trioctyl phosphine oxide (TOPO), trioctyl phosphine (TOP), and hexadecylamine (HDA) were purchased from Sigma-Aldrich Co., USA. DSPE-PEG 2000 {1,2-distearoyl-*sn*-glycero-3-phosphoethanolamine-N-[carboxy(polyethylene glycol)-2000]} and PEG-2-PE {1,2-palmitoyl-*sn*-glycero-3-phosphoethanolamine-N-[methoxy(polyethylene glycol)-2000]} were purchased from Avanti Polar Lipids, USA. Herceptin was obtained from Roche Pharma Ltd. (Basel, Switzerland). Cell culture reagents, fetal bovine serum (FBS), Dulbecco's modified eagle medium (DMEM, high glucose), penicillin-streptomycin, trypsin/EDTA, and Dulbecco's phosphate buffer saline (PBS) were supplied by Gibco BRL (Carlsbad, CA), and the SK-BR3 cells (breast cancer cells) were purchased from Korean Cell Line Bank.

### 2.1. Synthesis of CdSe/ZnS Core/Shell QDs

The synthesis of CdSe/ZnS quantum dots was performed using recently reported methods [27, 35]. A mixture of 9 mmol of trioctylphosphine oxide (TOPO), 7 mmol of tetradecyl phosphonic acid (TDPA), and 0.2 g of cadmium oxide (CdO) was heated to 240°C for 20 minutes to obtain a clear solution. A solution containing 0.01 g of Se powder dissolved in 5 mmol of trioctylphosphine (TOP) was injected quickly into the hot solution, and the reaction mixture was allowed to cool to 100°C for the growth of CdSe nanocrystals. To obtain the CdSe/ZnS nanoparticles, the solution was cooled to room temperature and highly luminescent CdSe nanocrystals were isolated and purified by centrifugation followed by precipitation with methanol and finally dissolved in 5 mL of toluene.

To obtain the CdSe/ZnS core-shell quantum dots, the precipitated CdSe nanocrystals were dispersed in 2 mL of TOP in a three-necked flask. In addition, ZnS (0.092 g) was dissolved in 2 mmol TOP upon gentle heating. After cooling to room temperature, the resulting mixture was injected dropwise into a reaction flask containing the core nanocrystal at 140°C for 6 hrs. After the addition was complete, the particles were annealed at 90°C for 6 hrs. Core-shell quantum dots of various sizes were obtained by adjusting the concentration of CdO and ZnS in TOP, as well as the corresponding injection periods. The prepared CdSe/ZnS QDs were dissolved in chloroform and purified further by centrifugation and double reprecipitation from methanol.

### 2.2. Preparation of Hydrophilic CdSe/ZnS QDs

DSPE-PEG and PEG-2 PE (2 : 8) were dissolved in 5 mL of chloroform (CHCl_3_) and 1 mL of the mixture was transferred to a 250 mL three neck round-bottom flask containing CdSe/ZnS QDs in 5 mL of chloroform. The clear solution was stirred overnight under nitrogen. When the reaction was complete, the chloroform was removed by vacuum, and the residue was mixed with 4 mL of water and transferred to a centrifuge tube. Subsequently, 40 mL of water was added to the mixed solution, and the precipitated product was separated by centrifugation (3,000 rpm for 15 min) and washed with water. The precipitated product was dissolved in 10 mL of methanol, and water was then added to ensure that polymer-coated QDs were suspended.

### 2.3. Immobilization of Herceptin on the Surface of CdSe/ZnS QDs

**Figure 1:**
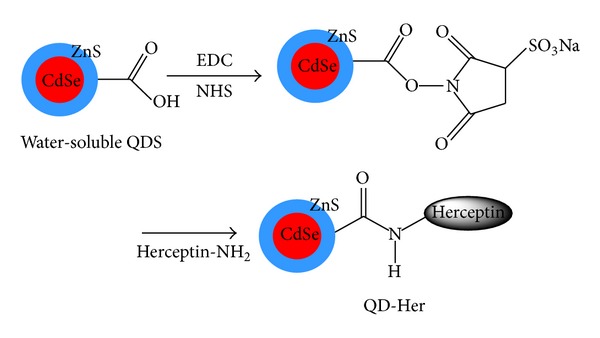
Schematic diagram showing the immobilization of herceptin on the CdSe/ZnS core-shell quantum dots (QD-Her).

The immobilization of herceptin on CdSe/ZnS (QD-Her) was carried out by a reaction of water-soluble QDs with herceptin, as shown in Figure 1. Water soluble QDs (18 mg/mL) and herceptin (108 mg/mL) were added to a two-necked round-bottom flask and dissolved in 10 mL of PBS buffer (pH 6.0). EDC (1 mmol) and NHS (1 mmol) were added to the reaction solution followed by stirring for 5 hrs at room temperature. The reaction solution was filtered to remove the precipitate and then added to a dialysis membrane (MWCO: 100,000) in deionized water media for 24 h to remove the unreacted EDC, NHS, and herceptin. Finally, the solution was filtered through a 0.45 *μ*m membrane and dried for 24 hrs under vacuum.

## 3. Results

### 3.1. Physical Characterization

Quantum dot nanoparticles were ground with KBr powder, compressed into pellets, and examined by FTIR (Jasco FTIR 300 E spectrometer) spectroscopy with a resolution of 4 cm^−1^. Transmission electron microscopy (TEM, Philips, CM 200 TEM, applied operation voltage; 120 kV) was used to observe the morphology of the nanoparticles. To obtain the samples for the TEM observations, the particles were diluted with distilled water and deposited on Formvar-coated 400 mesh copper grids. After drying the nanoparticle-fluid thin film on the copper grid, a thin carbon film, approximately 10–30 nm in thickness, was deposited on the nanoparticles fluid film. The hydrodynamic diameter and size distribution of the quantum dots were determined by dynamic light scattering (DLS) using a standard laboratory built light scattering spectrometer using a BI90 particle sizer (Brookhaven Instruments Corp., Holtsville, NY). The system had a vertically polarized incident light of 514.5 nm supplied by an argon ion laser (Lexel laser, model 95). The UV-Vis absorption spectrum was recorded from aqueous dispersions at room temperature using a Hitachi U-3000 spectrophotometer.

### 3.2. Cell Culture

SK-BR3 (breast cancer cells) was used as the target cell, and KB (epithelial cancer cells) was used as the control cell line. The cells were cultured routinely at 37°C in a humidified atmosphere containing 5% CO_2_ in a polystyrene dish containing 10 mL of McCOY medium or DMEM medium, supplemented with 10% fetal bovine serum and 1% penicillin streptomycin G sodium (PGS). The medium was changed every third day. For subculture, the cells were washed twice with PBS and incubated with a trypsin-EDTA solution (0.25% trypsin, 1 mM EDTA) for 10 min at 37°C to detach the cells. Complete media were then added to the polystyrene dish at room temperature to inhibit the effects of trypsin. The cells were washed twice by centrifugation and resuspended in complete media for reseeding and growing in new culture flasks. To observe the morphology of cells, the cells were seeded at a concentration of 1 × 10^5^/mL in a 10 mL Petri dish and incubated for 3 days with QDs-or QD-Her-containing media at a concentration of 0.2 mg/mL. The morphology of adhered cells was observed by optical microscopy (Nikon Eclipse TS100, Japan).

To examine the cellular uptake of nanoparticles via fluorescence microscope and confocal laser microscope, the cells were seeded at a concentration of 1 × 10^5^/mL in a 10 mL Petri dish and incubated for 1 day. After 1 day, the medium was replaced with QDs and QD-Her-containing media at a particle concentration of 50 *μ*g/mL and incubated for certain time (1–6 hrs) for the internalization of the nanoparticles into the cells. The cells were then washed three times with Dulbecco's PBS (D-PBS) and images were taken using fluorescence and confocal laser microscopes. The fluorescence images were obtained using an Olympus IX70 fluorescence microscope equipped with a cooled charge-coupled device (CCD) camera. The images were processed using DVC view software (version 2.2.8, DVC Company). A Zeiss LSM 410 confocal laser scanning microscope (Brightness: 700 cd/mm^2^, Zeiss, Oberkochen, Germany) was used to obtain the confocal images. The position and integrity of the internalized QD-Her conjugates were evaluated by confocal microscopy using 4,6-diamidine-2-phenylindole dihydrochloride (DAPI, blue) as a marker, which stains the nuclei of the cells. The cell nuclei were stained by the addition of DAPI solution (10 *μ*L) with proper mixing and incubated for 10 min. To track the QD-Her nanoparticles, herceptin-conjugated QDs and DAPI (488 nm) were added to the cells. The stained cells were washed at least three times with 1 mL of fresh McCoy medium and images were then taken by confocal laser microscopy [37].

The comparative proliferation of SK-BR3 and KB cells in a medium containing QDs and QD-Her was determined using a MTT (3-{4,5-dimethylthiazol-2yl}-2,5-diphenyltetrazolium bromide) assay. Briefly, the SK-BR3 and KB cells were seeded separately (1 × 10^5^ cell/mL) on 24 well plates in the presence of a cell culture medium. After 24 h, the culture medium was replaced with fresh medium containing QDs and QD-Her at a particle concentration of 200 *μ*g/mL. After incubation for 1, 2, and 3 days, a 50 *μ*L MTT solution (5 mg/mL in PBS) was added to each well and incubated in a humidified atmosphere of 5% CO_2_ at 37°C for 4 h. After removing the medium, the converted dye was dissolved in acidic isopropanol (0.04 N HCl-isopropanol) and kept for 30 min in the dark at room temperature. From each sample, the medium (100 *μ*L) was taken, transferred to a 96-well plate, and subjected to the ultraviolet measurements of the converted dye. This was achieved at a wavelength of 570 nm on a kinetic microplate reader. The experiment was repeated at least three times.

The phase contrast and fluorescence images of the cells were obtained using a combined explorer system with a motorized inverted fluorescence microscope (Carl Zeiss LSM700, Germany), using the topographic images that can be detected simultaneously. The cell proliferation experiment was performed in triplicate and the results were expressed as mean ± standard deviation (SD). Student's *t*-test was used to assess the statistical significant differences in the results. A *P* value <0.05 was considered significant.

## 4. Discussions

### 4.1. Surface Characterization of Herceptin-Immobilized QDs

**Figure 2:**
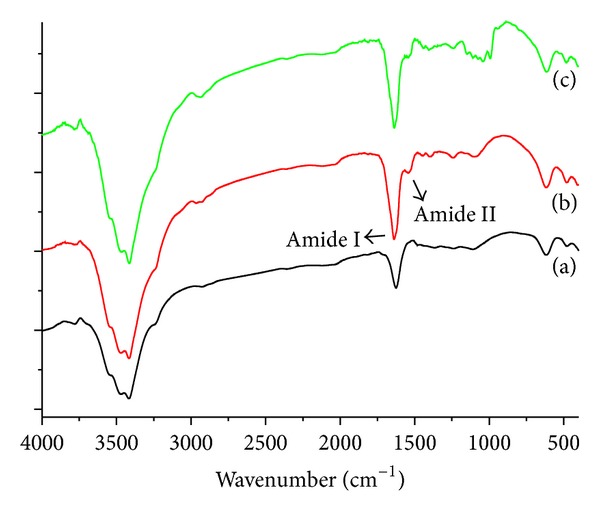
FT-IR spectra of the QDs (a), QD-Her (b), and herceptin (c) measured using the KBr method.

The surface modification of QDs with herceptin was confirmed by FTIR, as shown in Figure 2. In the case of the QDs spectrum, the introduction of DSPE-PEG 2000 to the surface of the QDs was confirmed by observing the characteristic peaks at 1700 and 3500 cm^−1^, as shown in Figure 2(a), which was attributed to free carboxyl (–COOH) and hydroxyl (–OH) groups [38, 39]. Again, after the reaction of the QDs with herceptin, two new peaks at positions approximately 1648 cm^−1^ and 1540 cm^−1^ were observed in the spectrum of QD-Her (Figure 2(b)), which were assigned to amide I (–CO–NH–) and amide II (–CO–NH–) bonds, respectively, indicating the successful immobilization of herceptin on the surface of the QDs [29, 32].

**Figure 3:**
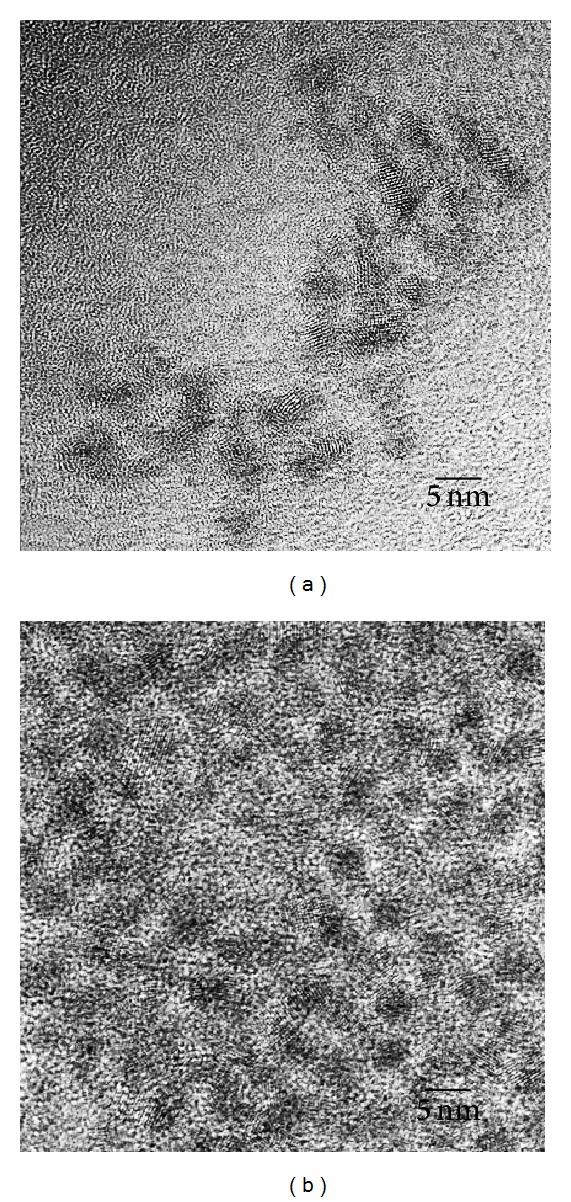
TEM images of the QDs (a) and QD-Her (b).

**Figure 4:**
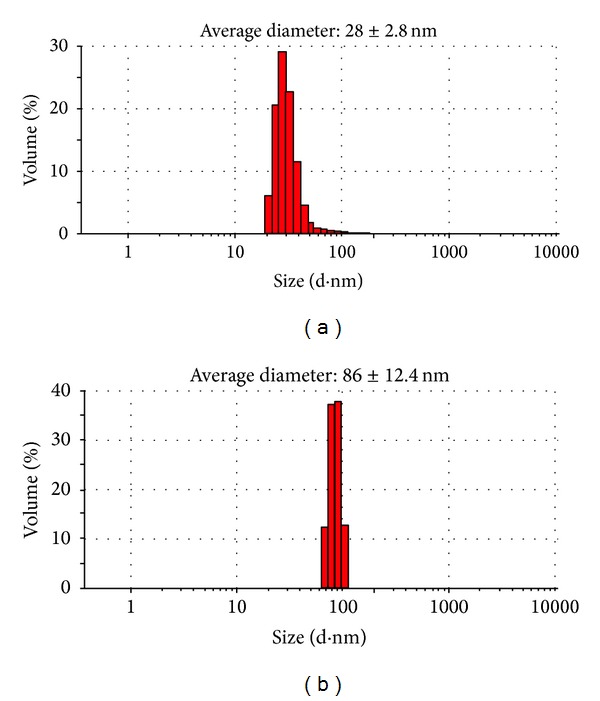
Particle size distribution of the QDs (a) and QD-Her (b) measured by dynamic light scattering (DLS).

**Figure 5:**
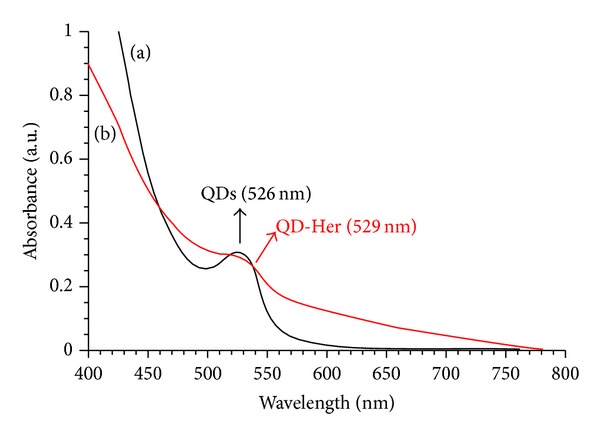
UV-Vis absorption spectra of the QDs (a) and QD-Her (b) in aqueous solution.

Figures 3(a) and 3(b) present TEM images of the QDs and QD-Her, respectively. The QDs have a spherical morphology with a mean diameter of ~4.1 nm. Because of the small dimensions and high surface energy of the particles, it was easy for them to aggregate, as seen in Figure 3(a). On the other hand, in the case of QD-Her, the particles had a mean diameter of 4.5 nm, were spherical in shape, and showed significantly less aggregation (Figure 3(b)). The larger particle size and nonaggregated particles morphology was attributed to the conjugation of herceptin on the surface of the QDs.  Figure 4 shows the typical size and size distribution of the synthesized QDs (Figure 4(a)) and QD-Her (Figure 4(b)) measured by DLS. The mean size of the QDs as determined by DLS was ~28 nm. On the other hand, the mean size of the QD-Her was approximately 86 nm. The particle size, as determined by DLS, was considerably larger than that determined by TEM. This is because the DLS technique measures the mean hydrodynamic diameter of the QDs core surrounded by the organic and solvation layers, and this hydrodynamic diameter is affected by the viscosity and concentration of the solution. TEM, however, gives the diameter of the core alone [29]. The synthesis of CdSe/ZnS core-shell QDs and herceptin-immobilized QDs was also confirmed by UV-Vis absorption spectroscopy, as shown in Figure 5. The QDs showed an absorption onset at 526 nm (Figure 5(a)) and after herceptin immobilization, it exhibited a red shift to 529 nm [31]. This red shift was caused by strong quantum confinement due to the increase in particle size. In addition, the peak at 529 nm attributed to the herceptin labels on the surface of QDs, because of metal to ligand charge transfer [34].

**Figure 6:**
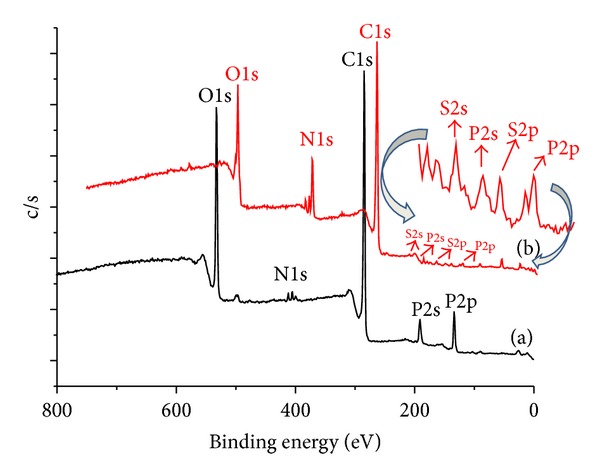
ESCA survey scan spectra of the QDs (a**)** and QD-Her (b).

The immobilization of herceptin on the surface of QDs was confirmed by ESCA, as shown in Figure 6. The QDs showed peaks for five elements corresponding to C1s (binding energy, 284.0 eV) and O1s (binding energy, 526.5 eV), P2s, 2p (binding energy, 197.0 eV, 132 eV), and N1s (binding energy, 397.0 eV), as shown in Figure 6(a). On the other hand, after herceptin immobilization, the QD-Her showed three typical peaks corresponding to C1s, O1s, and N1s. Table 1 lists the chemical compositions of the QDs and QD-Her, which were calculated from the ESCA survey scan spectra. In the case of the QD-Her, the carbon content (73.1%) was higher than in the QDs (69.3%). Furthermore, one new element, sulfur (0.4%), was observed on the surfaces of the QD-Her, and in the case of QD-Her, the nitrogen content increased from 0.7% to 10.5%, indicating the successful immobilization of herceptin on the surface of the QDs. One possible explanation for the reduction in the P2s, 2p, and S2p peaks is the photoelectrons with energy loss and the increase in the binding energy during immobilization with herceptin [40].

### 4.2. Evaluation of Cytotoxicity

**Figure 7:**
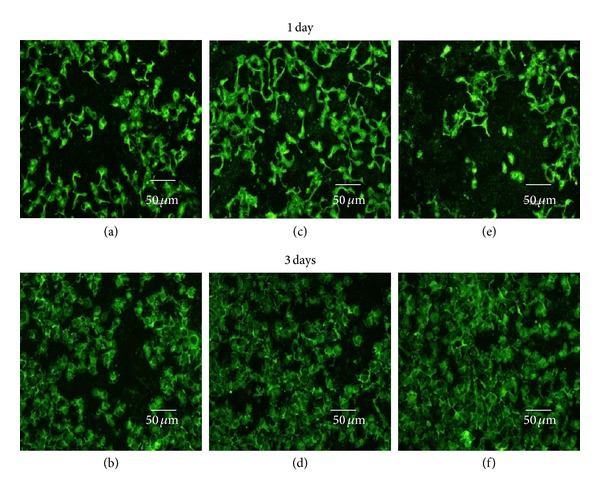
Fluorescence microscopy images of live and dead KB cells after culturing for 1 and 3 days in a polystyrene culture dish ((a), (b)) and in the presence of QDs ((b), (d)) and QD-Her ((e), (f)). The live and dead cells were stained and visualized in green and red, respectively, under a fluorescence microscope.

**Figure 8:**
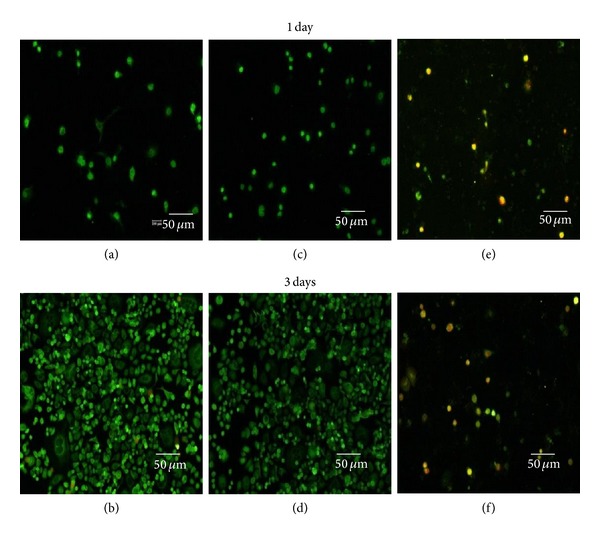
Fluorescence microscopy image of the live and dead SK-BR3 cells after culturing for 1 and 3 days in a polystyrene culture dish ((a), (b)) and in the presence of QDs ((b), (d)) and QD-Her ((e), (f)). Live and dead cells were stained in green and red, respectively, under a fluorescence microscope.

Figure 7 shows the status of the “Live/Dead” dye-stained SK-BR3 and KB cell cultured in the presence of QDs and QD-Her for 1 and 3 days of incubation. Using this qualitative method, the living and dead cells were stained in green and red under the fluorescence microscope, respectively. Figure 7 shows that all the KB cells remained viable after 3 days of incubation, irrespective of the presence or absence of nanoparticles. On the other hand, after a culture of 1 and 3 days, in the presence of QD-Her, most of the SK-BR3 cells had died, as shown in Figures 8(e) and 8(f). On the other hand, most of the SK-BR3 cells remained viable in the presence of the QDs and in the polystyrene culture dish (Figures 7(a)–7(d)), but a nonsignificant number of cells were dead in the QD-Her case. A possible explanation of this large decrease in cell viability in the case of QD-Her is that intracellularly delivered herceptin exhibits acute apoptotic activity by interacting with several transcription factors related to cell proliferation [39]. Previously, Bae et al. reported that degradable heparin nanogels and heparin/chitosan polyelectrolyte nanocomplexes could effectively induce apoptosis via receptor-mediated endocytosis through specific herceptin-HER2 integrin interaction [39]. The endocytosed QD-Her within the cells would release free herceptin molecules in the cytoplasm by cleaving the QD-herceptin linkage under the reductive intracellular environment, which has 300 times higher glutathione (GSH) concentration (20 Mm) than the extracellular level [41]. GSH is the most abundant reducing agent in the cytoplasm, facilitating the detachment of herceptin from the QDs by breaking the PEG-herceptin linkage. In addition, nanoparticles are taken up by the cells through endocytosis, which disrupts the cell membrane [32], or weak cell adhesive interactions with QDs promote apoptosis (programmed cell death). The core-shell nanoparticles conjugated with herceptin may act as cellular markers and target the receptors expressed on the cell surface with cellular internalization. The receptors are highly regulated by the cell surface proteins [37], which mediate the specific interactions between the cells and their extracellular milieu, and they are generally localized on the plasma membrane [33]. This suggests that the cytotoxicity of QDs was improved by the conjugation of DSPE-PEG and PEG-2-PE (Figures 7(c), 7(d)) and herceptin (Figures 7(e), 7(f)), as determined by the viability of KB cells (see Figure 7).

**Figure 9:**
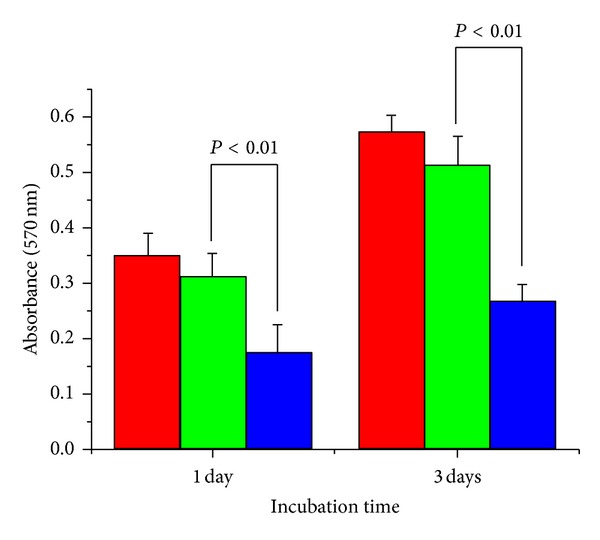
MTT assay, absorbance as a measure of the cell proliferation of SK-BR3 cells cultured in the PS culture dish (the red bar), in the presence of QDs (the green bar) and QD-Her (the blue bar) for different time.

Figure 9 shows the viability of SK-BR3 cells cultured for 1 and 3 days in the presence of QD-Her, as determined by the MTT assay. After 1 and 3 days of incubation, the level of SK-BR3 cell proliferation in the presence of QDs was similar to that of the cells cultured in the absence of nanoparticles (PS culture dish). On the other hand, cell proliferation in the presence of QD-Her was significantly lower than that of the QDs. Therefore, the CdSe/ZnS quantum dots conjugated with herceptin could increase the death of SK-BR3 cells considerably compared to the CdSe/ZnS quantum dots without herceptin.

### 4.3. Evaluation of Intracellular Uptake

**Figure 10:**
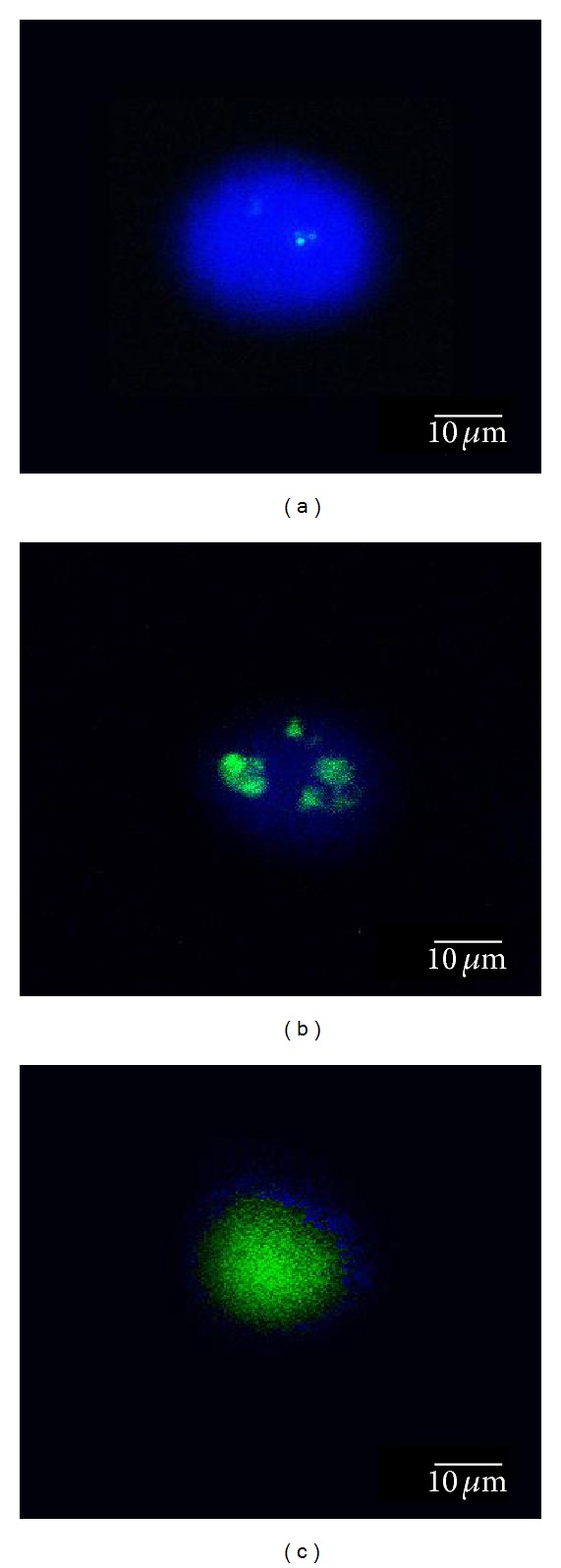
Fluorescence images obtained from the culture of SK-BR3 cells for 1, 3, and 6 hrs in the presence of DAPI and QD-Her.

The uptake of QD-Her into the target cells was visualized by fluorescence microscopy.  Figure 10 shows fluorescence images obtained from the cultured SK-BR3 cells that had been incubated for up to 6 hrs in the presence QD-Her. During the cell culture in the presence of QD-Her, a significant number of nanoparticles were transported into the cells and emitted intense fluorescence. This suggests that the QDs carrying herceptin provide specific recognition signals for the nanoparticles to facilitate internalization into the target cells (SK-BR3 cells). The interaction of the herceptin from the QDs with the HER-2 receptors expressed on the membrane surface of the SK-BR3 cells might have contributed to the improvement in the internalization of QD-Her into the cells, based on receptor-mediated endocytosis [29]. Gan et al. reported similar results [42]. They introduced a hepatocarcinoma binding peptide (A54) onto the surface of the magnetite nanoparticles and examined their interaction with hepatocellular carcinoma cells *in vitro* by fluorescence microscopy. Internalization of the herceptin-conjugated nanoparticles (QD-Her) into SK-BR3 occurred. Breast cancer cells expressing HER-2 receptor were quite sensitive to herceptin.  Figure 10 shows that herceptin is an effective antibody, binding specifically to the HER-2 receptor-bearing breast cancer cells. The internalization of QD-Her into SK-BR3 cells was confirmed by confocal laser microscopy to characterize the delivery of QD-Her to the cytoplasm of the SK-BR3 cells.  Figure 10 shows the fluorescence image derived from the nucleus of the SK-BR3 cells (DAPI, blue) and QD-Her internalized (green). The cells were cultured in the presence of QD-Her at various incubation times (Figure 10). Weak QD-Her conjugates were observed in the fluorescence image (green color) after 1 hr (Figure 10(a)) and slightly higher fluorescence image was observed after 3 hrs (Figure 10(b)). Intense fluorescence image was noted after 6 hrs (Figure 10(c)). On the other hand, the blue fluorescence image derived from the nuclei stained with DAPI was strong after 1 hr incubation but it decreased with increasing incubation time and almost disappeared after 6 hrs incubation. In particular, the interaction of SK-BR3 with QD-Her began after 1 hr incubation and was accelerated and saturated after 6 hrs. The confocal microscopy images suggest that the nanoparticle-mediated delivery of monoclonal antibodies was achieved efficiently, resulting in cell death. The mechanism of internalization involves endocytosis followed by the release of herceptin-conjugated nanoparticles to the cytoplasm [37]. This suggests that the growth signal of breast cancer cells is inhibited completely by the specific binding of the herceptin to the Her-2 receptor of SK-BR3 membrane, resulting in cell death [38].

## 5. Conclusions

DSPE-PEG-coated CdSe/ZnS core-shell quantum dots (QDs) were conjugated successfully with the herceptin antibody. Herceptin immobilized QDs (QD-Her) were confirmed by FTIR and XPS. The QD-Her size determined by DLS was ~86 nm. The QD-Her had no cytotoxicity on the control cells (KB) compared to the target cells (SK-BR3). QD-Her was internalized selectively into the target cells (SK-BR3), and free herceptin was released in the cytoplasm, which induced acute apoptosis. The QD-Her nanoparticles were endocytosed by breast cancer cells (SK-BR3) to a large extent via a receptor-mediated mechanism, where herceptin conjugated on the nanoparticles targets the HER-2 receptor expressed on the membrane of the cancer cells [39]. Therefore, QD-Her has a potential use in optical imaging and the treatment of breast cancer cells.

## Figures and Tables

**Table 1:** Atomic percentage of QDs and QD-Her calculated from the ESCA survey scan spectra.

Substrates	Atomic (%)			
	C	O	N	P
QDs	69.3	24.0	0.7	6.0

QD-Her	73.1	15.5	10.5	0.5
